# Risk Factors of Hypertension among Diabetic Patients from Yaoundé Central Hospital and Etoug-Ebe Baptist Health Centre, Cameroon

**DOI:** 10.1155/2020/1853516

**Published:** 2020-06-15

**Authors:** Beryl Kemche, Brice Ulrich Saha Foudjo, Elie Fokou

**Affiliations:** ^1^Department of Biochemistry, University of Yaoundé I, PO Box 337, Yaoundé, Cameroon; ^2^Department of Biochemistry, University of Bamenda, PO Box 39, Bambili, Cameroon

## Abstract

Uncontrolled blood pressure is a threat to diabetic patients' life. The aim of this study was to identify risk factors of hypertension among diabetic patients at different stages from Yaoundé Central Hospital and Etoug-Ebe Baptist Health Center of Cameroon. A hospital-based cross-sectional study was conducted for 6 months, and 109 participants (types 1 and 2), aged 24–81 years, were enrolled using simple random sampling. A pretested structured questionnaire was used to collect sociodemographic data, habitual behaviors, clinical history blood pressure, and anthropometric measures. The prevalence of hypertension was 86.2%. Of the total, 13.8% participants were normotensive, 32.1% stage 1 hypertensive, and 54.1% stage 2 hypertensive. Being a male (*p* = 0.046) and not smoking (*p* = 0.036) were negatively associated with stage 1 hypertension whereas eating less than 3 times (*p* = 0.046) and duration of diabetes greater than 9 years among women (*p* = 0.039) were positively associated. Age above 40 years (*p* = 0.002) was negatively associated with stage 2 hypertension. However, age above 40 years had a negative effect among Christian, less educated diabetics, people having diabetes for more than 9 years, and those on medical treatment (5.556 ≤ specific OR ≤ 10.278). Duration of diabetes (age-adjusted OR = 1.155; *p* = 0.003) and abnormal waist circumference (crude OR = 4.074; *p* = 0.024) were positively associated with stage 2. Abnormal waist-to-hip ratio (crude OR = 3.773; *p* = 0.028) and feeding rate greater than 2 times a day (WHR-adjusted OR = 3.417; *p* = 0.046) were positively associated with hypertension (stages 1 and 2). This study suggests that hypertension, present at its two stages, is a serious health issue among diabetic patients. Thus, appropriate intervention should be put in place to prevent and control hypertension by managing identified risk factors.

## 1. Introduction

Hypertension is becoming a serious concern in low- and middle-income countries based on the statistics. The prevalence of high blood pressure in sub-Saharan African ranges between 14.5% in rural Eritrea [1], 32.9% in semiurban Ghana [2], and 40.1% in urban South Africa [3]. In Cameroon, hypertension is still uncommon in rural Cameroon but occurs frequently in urban community. The prevalence spans from 5.7% in rural settings [4] to 47.5% in urban milieu [5], with a national average survey of 31.0% [6]. The burden of hypertension is forecast to increase by 2025 as it is predicted to reach a total of 1.56 billion worldwide [7]. This rise is clearly associated with the adoption of Western lifestyles by people in developing countries. The increase of morbidity and mortality from cardiovascular diseases related to hypertension is expected to follow [8–10].

Recent studies carried out worldwide have shown that the expected rise is also related to the scourge in the prevalence of diabetes among other risk factors [7, 11]. Epidemiologic studies have depicted that hypertension occurrence was 1.5-2 times greater among diabetic patients than in an approximately matched nondiabetic cohort [7, 12, 13]. Thus, managing hypertension among diabetics would lead to a drastic reduction of its incidence.

Several hypotheses were conjectured to explain the pathogenic relationship between hypertension and diabetes mellitus, and these include (i) insulin resistance produces stimulatory effects on the sympathetic nervous system and the renin-angiotensin system and (ii) abnormalities in catecholamines and sodium metabolism [14–16]. Nevertheless, hypertension is asymptomatic and is usually diagnosed incidentally or after major organ damage has occurred [17].

Independent risk factors for hypertension in the general population are known, among others, to be age, low educational status, being married, obesity, high dietary salt intake, low dietary intake of calcium and potassium, alcohol consumption, psychosocial stress, low levels of physical activity, and family history of hypertension [18–22]. So far, few risk factors of hypertension have been highlighted among diabetics in some countries (India, Nigeria, Benin, and United Arab of Emirates), namely, age above 55 years, abdominal obesity, and duration of diabetes [23–26]. Duration in diabetes was found to be the most common.

The epidemiology and etiology of hypertension in Cameroon are not well investigated, particularly among diabetics where no study has been carried out so far. From a study conducted in the USA, Berlowitz et al. [27] reported that diabetic patients received less intensive antihypertensive medication therapy than patients without diabetes. Thus, the main aim of this study was to identify risk factors among diabetics visiting Yaoundé Central Hospital and Etoug-Ebe Baptist Health Center. It will provide further information regarding the burden of hypertension and its risk factors to inform health policies and plan further interventions.

## 2. Materials and Methods

### 2.1. Clinical Settings

The study was conducted at the Yaoundé Central Hospital and the Etoug-Ebe Baptist Health Center, both located in the capital city of Cameroon. These two health centers were chosen as they have diabetes units. The work was conducted in these two health centers due to the scarcity of diabetic patients in Cameroon (prevalence of 4.3%). The Yaoundé Central Hospital is Cameroon's largest hospital and a 381-bed tertiary level general teaching hospital. It employs nearly 800 staff and possesses the National Center for Hypertension and Diabetes. The Etoug-Ebe Baptist Health was the first CBC health facility in the Francophone Cameroon. It serves at least 350 patients daily.

### 2.2. Study Design and Period

A hospital-based cross-sectional study was conducted between 01 June 2011 and 30 November 2011 to determine the hypertension's prevalence and its risk factors among diabetic outpatients.

### 2.3. Selection of Participants

As no study exists on hypertension among diabetic patients in Cameroon; the prevalence of diabetes in Cameroon was used to calculate the sample size. Considering a prevalence of 4.3%, a 95% confidence intervals (*z* = 1.96), a precision of 5%, and a nonresponse rate of 10%, the sample size is 72 diabetic individuals. In the end, 109 eligible and consenting candidates were recruited using a simple random sampling.

### 2.4. Inclusive Criteria

All patients with either type 1 or type 2 diabetes and hypertensive or not were included in the study. They were all greater than 20 years old.

### 2.5. Data Collection

A structured questionnaire that included general information on sociodemographic characteristics (marital status, gender, religion, education level, age, and profession), habitual behaviors (sport activity, sport duration, feeding rate, alcohol, and smoking intake), clinical history (duration in diabetes, type of diabetes, family history of diabetes, dietary treatment, and medical treatment), and anthropometry (waist-to-hip ratio, waist circumference, BMI, and systolic and diastolic blood pressures) was used for data collection. The new classification of blood pressure was used based on the 2017 ACC/AHA/AAPA/ABC/ACPM/AGS/APhA/ASH/ASPC/NMA/PCNA Guideline [28]: normal blood pressure (systolic blood pressure (SBP) <120 mmHg and a diastolic blood pressure (DBP) of <80 mmHg), elevated blood pressure (120 mmHg ≤ SBP ≤ 129 mmHg and DBP < 80 mmHg), hypertension stage 1 (130 mmHg ≤ SBP ≤ 139 mmHg or 80 mmHg ≤ DBP ≤ 89 mmHg), and hypertension stage 2 (SBP ≥ 140 mmHg or DBP ≥ 90 mmHg). Body mass index (BMI), waist-hip ratio, and waist circumference were measured and classified in accordance with the WHO guidelines [29].

### 2.6. Parameter Measurements

BP was measured using a mercury sphygmomanometer (adult size) by the auscultatory method. Blood pressure was taken while the patient was in a sitting position, from the right arm after the patient rested for at least 5 minutes before measurement. Two readings were taken at a 5 min interval, and the average of the two readings was reported. In case of a difference of >5 mmHg in the readings, two more readings were taken in a similar manner, and the average of all readings was reported. The point at which the first Korotkoff sound was heard was taken as systolic blood pressure (SBP), and the diastolic blood pressure (DBP) was taken to be the point at which the sound disappeared.

Weight and height were measured with participants standing without shoes and wearing light clothing. Weight was measured using a digital weighing scale. The scale was calibrated to the zero level before each measurement and was tested for repeatability of the measures. Height was measured by using a stadiometer while the patient was in an upright position.

Waist and hip circumferences were measured by using a flexible tape meter at both the level just above the iliac crest and at the maximum circumference of the hip, respectively.

### 2.7. Data Analysis

The data were entered and cleaned in MS Excel 2010 and exported to IBM/SPSS Windows version 20 (IBM Corporation, Armonk, NY, USA). Continuous variables were summarized as mean ± standard error (SBP and DBP) while categorical variables were summarized as frequencies and proportions. Chi-square was used to analyze the associations between different qualitative variables. An unpaired *t*-test was used to compare two means. A two-tailed *z*-test was employed to compare two proportions. To determine the risk factors associated with hypertension, bivariate and multivariate logistic regression analyses were carried out. Bivariate odds ratio estimation was followed by derivation of different multivariate models through logistic regression with forward entry and forward step-wise methods, and the best multivariate derived model was selected. This approach was used to adjust the odds ratios for confounders. *p* < 0.05 was considered statistically significant.

### 2.8. Data Quality Control

Surveyors were trained for 1 day, and a presurvey was conducted on 14 eligible diabetic patients to ensure consistency, reliability, and to reduce intra- and interobserver variation. Supervision was done by the main investigators throughout the data collection and analysis.

### 2.9. Ethical Consideration

Ethical clearance, Authorization No. 232/CNE/SE/2011, was obtained from the Cameroon Ethics Committee. Additional authorization was obtained from both the National Center for Hypertension and Diabetes of the Yaoundé Central Hospital and the Cameroon Baptist Convention Health Board of Etoug-Ebe Baptist Health Center. Written informed consent was obtained from participants after comprehensive explanation of the scope and procedure of the study in English or French when necessary. The anonym of participants was kept for an indefinite period of time.

## 3. Results

### 3.1. Socio-Demographic Characteristics

A total 109 participants were enrolled in the study and among them, 60.6% were women and 39.4% men. The age range was from 24 years to 81 years, with a mean age of 55 ± 11 years. Nearly nine-tenths (88.1%) of the participants was older than 40 years. Three-fourths of the participants were married (74.3%), 21.1% were widowed, and 4.6% were single. Approximately nine-tenths (89.9%) was Christian and 10.1% Muslim. Nearly two-thirds had a secondary education level (61.5%), 22.0% were illiterate, and 16.5% a postsecondary education level.

### 3.2. Clinical Characteristics

Nearly four-fifths (81.7%) of the participants had type 2 diabetes, and 18.3% had type 1 diabetes. The duration of diabetes in this study varied from few months to 29 years. Seventy-five participants (68.8%) have been having diabetes for less than 10 years and 34 participants (31.2%) for more than 9 years. Sixty-two participants (56.9%) had at least one parent that was diabetic, and forty-seven participants (43.1%) had no family history of diabetes. Nearly 40.4% of the participants were overweight, 33.0% were obese, and 25.7% had a normal weight. From the 109 participants, 87.2% of the participants had abnormal waist circumference, and 12.8% had a normal waist circumference. Seventeen participants out of twenty (85.3%) had an abnormal waist-to-hip ratio, and three participants out of twenty (14.7%) had a normal waist-to-hip ratio. Of the total, 83.5% of the participants were on dietary treatment, and 92.7% were on medical treatment. The rate of consumption of alcohol in the studied population was distributed as follows: rarely (32.1%), moderately (56.9%), and highly (11.0%). The rate of smoking was distributed as follows: never (92.7%), moderately (4.6%), and highly (2.8%). The daily feeding rate was distributed as follows: 1 to 2 times (33.0%), 3 to 4 times (64.2%), and more than 4 times (2.8%). In this population, 77.1% practiced sport as physical activity, and 22.9% did not. The average duration of sport was 49 ± 5 min.

Even though all the study population was on dietary treatment and/or medical treatment, no improvement of the BMI (Phi = 0.036, *p* = 0.933), waist-to-hip ratio (Phi = −0.001, *p* = 0.996), alcohol consumption (Phi = 0.113, *p* = 0.500), smoking rate (Phi = 0.137, *p* = 0.360), waist circumference (Phi = 0.022, *p* = 0.821), and feeding rate (Phi = 0.052, *p* = 0.864) was observed over time (duration in diabetes).

### 3.3. Pattern of Hypertension among Diabetics

Of the total, the mean value of the systolic blood pressure was 138 ± 21 mmHg, and the mean value of the diastolic blood pressure was 85 ± 11 mmHg. No significant difference was observed in the mean values of SBP (*t* = 0.403, *p* = 0.689) and DBP (*t* = −0.319, *p* = 0.750) of hypertensive diabetics in both genders. Age had no effect on the mean value of DBP (*t* = −1.347, *p* = 0.181) and had a significant effect on the mean value of SBP (*t* = −4.162, *p* ≤ 0.001). Participants older than 40 years old (140 ± 2 mmHg) had a high value of SBP when compared to those not older than 40 years old (125 ± 3 mmHg).

Among the study population, 13.8% were normotensive, 32.1% stage 1 hypertensive, and 54.1% stage 2 hypertensive. The prevalence of hypertension among diabetics was 86.2%. There was no statistical difference of prevalence of hypertension among women, 81.8%, and men, 93.0%, (*z* = 1.66; *p* = 0.097). There was no statistical difference of hypertension prevalence among type 1 diabetics, 85%, and type 2 diabetics, 86%, (*z* = −0.178; *p* = 0.859). The age range (Chi = 6.639, *p* = 0.036) was statistically significant among the hypertension categories (Table 1). Stage 2 hypertensive diabetics were older than normotensive diabetics with both having equal number of old people as stage 1 hypertensive diabetics. The sex (Chi^2^ = 2.988, *p* = 0.224) did not significantly change in each hypertension category (Table 1).

### 3.4. Risk Factors of Hypertension among Diabetics

Odds ratios were calculated for risk factors found to be associated with hypertension at different stages among diabetics (Table 2). Four risk factors were associated with stage 1 hypertension occurrence among normotensive diabetics: gender (*p* = 0.046), feeding rate (*p* = 0.046), duration of diabetes (*p* = 0.039), and smoking status (*p* = 0.036). Three risk factors were identified that caused the development of stage 2 hypertension among stage 1 hypertensive diabetics: age (*p* = 0.002), duration of diabetes (*p* = 0.003), and waist circumference (*p* = 0.024). The overall development of hypertension (stages 1 and 2) was governed by three risk factors: waist-to-hip ratio (0.028), smoking status (*p* = 0.043), and feeding rate (*p* = 0.046).

The results showed that being a male decreased the probability by 94.2% (adjusted OR = 0.058; 95% CI 0.004-0.956) of developing stage 1 hypertension among normotensive diabetics. When exploring risk factors in each gender using univariate analysis, smoking status (*p* = 0.035) and feeding rate (*p* = 0.027) were the influencing risk factors in men whereas duration of diabetes (*p* = 0.038) was the one in women.

Eating more than 2 times a day increased the risk by 6.478 (95% CI 1.035-40.533) of developing stage 1 hypertension. Not smoking decreased the probability by 98.3% (adjusted OR = 0.017; 95% CI 0.000-0.769) of developing stage 1 hypertension. Surprisingly, long duration of diabetes seemed to decrease the probability of developing stage 1 hypertension. However, by stratifying this association with gender, it was observed that the risk of developing stage 1 hypertension among normotensive females with more than 9 years of diabetes was 5.143 times (95% CI 1.033-25.602) higher than those with less than 10 years of diabetes. Among male, the association was nonsignificant (*p* = 0.943).

Stage 1 hypertensive diabetics with an age above 40 years old were 0.916 times (95% CI 0.866–0.969) more likely to become stage 2 hypertensive patients than those younger than 41 years old. When stratifying separately this association with the other 18 variables, duration of diabetes less than 10 years (*p* = 0.006), being on medication treatment (*p* = 0.023), education level less than First School Leaving Certificate (FSLC) (*p* = 0.015), and being Christian (*p* = 0.027) were found to be significant. Independent stratification with duration of diabetes less than 10 years, being on medication treatment, having an education level less than FSLC, and being Christian increased the risk of developing stage 2 hypertension among stage 1 hypertensive patients above 40 years old by 7.167 (95% CI 1.557-32.981), 5.778 (95% CI 1.091-30.584), 10.278 (95% CI 1.117-94.601), and 5.556 (95% CI 1.049-29.434), respectively. Thus, duration of diabetes less than 10 years, being on medication treatment, and having an education level less than FSLC were negative confounders.

Age-adjusted odds ratio of duration of diabetes showed that for every 1 year of diabetes, the odds of becoming stage 2 hypertensive patients increased by 1.155 (95% CI 1.051–1.270) among stage-1 hypertensive diabetics. Abnormal waist circumference among stage-1 hypertensive diabetics increased the risk by 4.074 (95% CI 1.126-14.735) of developing stage 2 hypertension.

The examination of the determinants that allowed to shift from normotensive diabetics to hypertensive diabetics (stages 1 and 2) gave the following results: waist-to-hip ratio (*p* = 0.028), smoking status (*p* = 0.043), and feeding rate (*p* = 0.046) were associated with the risk of developing hypertension. Normotensive diabetics with abnormal waist-to-hip ratio were 3.773 times (95% CI 1.087–13.091) more likely to develop hypertension than those with a normal waist-to-hip ratio. Using a univariate analysis, smoking seemed to reduce the risk of developing hypertension by 77.5%. However, after adjusting for waist-to-hip ratio, smoking was found to be nonsignificant. Normotensive diabetics, eating at least 3 times a day, were 3.417 times (95% CI 1.019-11.455) more likely to develop hypertension than those eating at most 2 times a day.

## 4. Discussion

The prevalence of hypertension was high among diabetics (86.2%) in the two health centers. However, lower prevalence rates of 54.2% and 70.4% were reported among Nigerian and Moroccan diabetic populations, respectively [30, 31]. One of the factors that could explain these lower prevalence rates may be the difference in the chosen cut-off point for high blood pressure selected by the authors, ≥140/90 mmHg. In this study the cut-off point was based on the new guidelines, which is ≥130/80 mmHg. If the same cut-off point was used (≥140/90 mmHg), the prevalence of hypertension of this study would have been 54.1%.

Among the study population, stage 2 hypertensive patients had the highest prevalence, followed by stage 1 hypertensive and normotensive patients. A reverse pattern was observed by Venugopal and Mohammed [32] among type 2 diabetics: 52.4% patients were prehypertensive, 18% patients were in stage 1 hypertension, and 7.6% had stage 2 hypertension.

Modifiable and nonmodifiable risk factors were identified at different stages of hypertension in this study. Under modifiable risk factors, there were waist-to-hip ratio, waist circumference, smoking status, and feeding rate. Age, gender, and duration of diabetes were the nonmodifiable risk factors.

When looking at the evolution of hypertension, age was found to influence only the development of stage 2 hypertension from stage 1 hypertensive diabetics. Its overall effect showed that getting older seemed to have protective effect against stage 2 hypertension. This overall effect was found to be disjointedly confounded by duration of diabetes less than 10 years, being on medication treatment, having an education level less than First School Leaving Certificate (FSLC) (5.556 ≤ specific odds ratios ≤ 10.278). These specific odds ratios were more consistent with the findings of Tabi Arrey et al. [22] that observed in the general population of Cameroon that age above 40 years increased hypertension risk by 4.5 (95% CI 3.0-6.8). In Benin, age above 55 years was positively associated with increasing risk of hypertension among diabetics using a bivariate analysis (OR = 6.1, *p* ≤ 0.001) [24]. In fact, the vascular system changes with age due to the alteration and relative reduction of elastic fibers that are replaced by collagen tissue in artery wall. This evolution induces more rigidity of arteries contributing to elevate blood pressure [24]. Moreover, with age, there is a decrease of baroreceptor sensitivity, an increase of responsiveness to sympathetic nervous system stimuli, an alteration of renal and sodium metabolism, and a modification of renin-aldosterone relationship, thereby predisposing to high blood pressure [33].

Abnormal waist circumference (crude OR = 4.074) was associated with stage 2 hypertension. Similar results, with different intensity, were obtained in Benin [24]. The waist circumference (abdominal obesity) was positively associated (OR = 1.9, *p* = 0.036) with the risk of hypertension among Beninese diabetics. This suggested that diabetics with abdominal obesity had a high predisposition of developing stage 2 of hypertension. The mechanism is not entirely understood. However, research emphasized the major role of increased sympathetic activity in obesity-hypertension. Long-term sympathoactivation could raise arterial pressure by triggering peripheral vasoconstriction and by increasing renal tubular sodium reabsorption. Recent evidence indicates that leptin may represent a connection between excess adiposity and increased cardiovascular sympathetic activity [34].

Gender was observed to foster stage 1 hypertension among normotensive diabetics. Male diabetics had a low probability than female ones. However, Kingue et al. [6] showed that in the general population of Cameroon, males had an increased hypertension risk of 1.23 (95% CI 1.142-1.331) compared to females. This conclusion was also reached by many other researchers. This might either mean that in diabetes, women are more exposed to hypertension than men or the effect of duration of diabetes in women in this study had an important influence on hypertension occurrence compared to smoking and feeding rate in men as pointed out by the results.

Abnormal waist-to-hip ratio was positively associated with hypertension (both stages 1 and 2) among diabetics. This finding was also highlighted by Picon et al. [35] whose work showed that waist-to-hip ratio was associated with hypertension risk among Portuguese diabetics. In this study, BMI was not found to be a significant risk factor. Thus, this reiterated the fact that diabetics seem to be inclined to develop abdominal obesity as observed by Yadav et al. [36] with an increased risk of 1.56 (95% CI 1.37-1.79) in the Indian population.

Not smoking decreased the risk of getting stage 1 hypertension among normotensive diabetics. Even though available data did not portray a direct causal relationship, Virdis et al. [37] believed that cigarette smoking associated with affecting arterial stiffness and wave reflection could have greater adverse effect on central blood pressure. Hypertensive smokers are more predisposed to develop severe forms of hypertension, including malignant and renovascular hypertension. This might be due to an accelerated atherosclerosis.

Feeding rate had the highest influence (adjusted odds ratios of 3.417 and 6.478) on hypertension compared to the other significant risk factors. In reality, it is dietary sodium daily intake that causes elevation of blood pressure while dietary potassium intake reduces hypertension risk [38]. Thus, in this study, increasing the feeding rate might be strongly related to the increase of dietary sodium.

Duration of diabetes was the only risk factor present at every stage of hypertension. Its impact was greater between stage 1 hypertensive diabetics and stage 2 hypertensive diabetics (adjusted OR = 1.115) than between normotensive diabetics and stage 1 hypertensive diabetics (adjusted OR = 0.856). In one study conducted in Nigeria, duration of diabetes was shown to be correlated to hypertension (*p* = 0.04) among type 2 diabetics [26]. In another study conducted in India, the prevalence of hypertension was shown to increase with the duration of diabetes [23]. This result may be related to chronic hyperglycemia resulting in endothelial suffering leading to thickening of the arterial wall and to a rise in blood pressure later [24].

Even though this survey has come up with important findings regarding the risk factors of hypertension among diabetics, there are certain limitations that should be pointed out. First of all, there was possibility that patients underreported their family history of diabetes, which was based on only self-reports. Secondly, the study involved type 1 and type 2 diabetics which may exhibit different characteristics that may distort relationship between hypertension and its actual risk factors. The fact that this was a hospital-based cross-sectional study indicates that this study may lack generalizability to the entire population. Finally, the small sample size used in this study compared to other studies may be unable to reflect the real effect of and identify all the risk factors.

## 5. Conclusion

The present study showed that hypertension has a high magnitude (prevalence of 86.2%) among diabetics from the Yaoundé Central Hospital and the Etoug-Ebe Baptist Health Center. It also highlighted the fact that the nature of significant risk factors changed with hypertension evolution, except duration of diabetes that was present at each stage. Modifiable risk factors were waist-to-hip ratio, waist circumference, smoking, and feeding rates. Nonmodifiable risk factors were gender, age, and duration of diabetes. Thus, special care should be given to patients with more than 9 years of diabetes. Likewise, early identification of normotensive diabetics consuming sodium-rich diets, especially women, and/or smoking should be a priority. Among stage 1 hypertensive diabetics, care should be emphasized on patients older than 40 years and/or with abnormal waist circumference. Moreover, Christian and less educated diabetics should be sensitized on diabetes management. These findings also indicate the importance of carrying similar studies in different geographical regions of Cameroon to delineate the exact pattern of hypertension among diabetic patients across the country.

## Figures and Tables

**Table 1:** Age- and gender-wise distribution of diabetic patients based on their systolic and diastolic blood pressure.

Factors	Normotensive	Stage 1 hypertensive	Stage 2 hypertensive
Age (years)			
>40 years	11^a^ (73.3%)	29^ab^ (82.9%)	56^b^ (94.9%)
≤40 years	4 (26.7%)	6 (17.1%)	3 (5.1%)
Statistics	Chi^2^ = 6.639; *p* = 0.036		
Gender			
Female	35 (36.4%)	19 (54.3%)	12 (80.0%)
Male	24 (63.6%)	16 (45.7%)	3 (20.0%)
Statistics	Chi^2^ = 2.988; *p* = 0.224		

Frequencies carrying the same superscript letter on the same row were statistically significant (*p* < 0.05).

**Table 2:** Risk factors at different hypertension stages among diabetic participants.

Predictor variables	Hypertensive stage 1 (*n* = 35)	Normotensive (*n* = 15)	Univariate analysis		Multivariate analysis	
	*N* (%)	*N* (%)	OR (95% CI)	*p* value	OR (95% CI)	*p* value
Gender						
Male	16 (45.7%)	3 (20.0%)	1 (reference)		0.058 (0.004-0.956)	0.046^∗^
Female	19 (54.3%)	12 (80.0%)	0.297 (0.071-1.240)	0.086	1 (reference)	
Feeding rate						
≥3 times a day	23 (65.7%)	7 (46.7%)	2.190 (0.639-7.504)	0.208	6.478 (1.035-40.533)	0.046^∗^
<3 times a day	12 (34.3%)	8 (53.3%)	1 (reference)		1 (reference)	
Duration of diabetes						
≥10 years	17 (48.6%)	4 (26.7%)	2.597 (0.692-9.747)	0.150	0.856 (0.739-0.992)	0.039^∗^
<10 years	18 (51.4%)	11 (73.3%)	1 (reference)		1 (reference)	
Smoking status						
Yes	2 (5.7%)	3 (20.0%)	0.242 (0.036-1.633)	0.123	0.017 (0.000-0.769)	0.036^∗^
No	33 (94.3%)	12 (80.0%)	1 (reference)		1 (reference)	
Occupation						
Not retired or not a housewife	21 (60.0%)	5 (33.3%)	1 (reference)		0.435 (0.080-2.361)	0.335
Retired or housewife	14 (40.0%)	10 (66.7%)	0.333 (0.094-1.185)	0.084	1 (reference)	
Tribe						
Not from the west region	18 (51.4%)	8 (53.3%)	1.079 (0.321-3.626)	0.902	0.257 (0.039-1.699)	0.159
West region	17 (48.6%)	7 (46.7%)	1 (reference)		1 (reference)	

From stage 1 to stage 2 in hypertension	Hypertensive stage 2 (*n* = 59)	Hypertensive stage 1 (*n* = 35)				

Age						
>40 years	56 (94.9%)	29 (82.9%)	3.862 (0.900-16.574)	0.055	0.916 (0.866-0.969)	0.002^∗^
≤40 years	3 (5.1%)	6 (17.1%)	1 (reference)		1 (reference)	
Duration of diabetes						
≥10 years	13 (22.0%)	17 (48.6%)	0.299 (0.121-0.739)	0.008^∗^	1.155 (1.051-1.270)	0.003^∗^
<10 years	46 (78.0%)	18 (52.4%)	1 (reference)		1 (reference)	
Waist circumference						
Abnormal	55 (93.2%)	27 (77.1%)	4.074 (1.126-14.735)	0.024^∗^	1.005 (0.965-1.047)	0.802
Normal	4 (6.8%)	8 (22.9%)	1 (reference)		1 (reference)	

Normotensives to hypertensives	Hypertensive (*n* = 94)	Normotensive (*n* = 15)				

Waist-to-hip ratio						
Abnormal	83 (88.3%)	10 (66.7%)	3.773 (1.087-13.091)	0.028^∗^	0.000 (0.000-0.007)	0.003^∗^
Normal	11 (11.7%)	5 (33.3%)	1 (reference)		1 (reference)	
Smoking status						
Yes	5 (5.3%)	3 (20.0%)	0.225 (0.048-1.062)	0.043^∗^	0.175 (0.031-1.008)	0.051
No	89 (94.7%)	12 (80.0%)	1 (reference)		1 (reference)	
Feeding rate						
≥3 times a day	66 (70.2%)	7 (46.7%)	2.694 (0.891-8.146)	0.072	3.417 (1.019-11.455)	0.046^∗^
<3 times a day	28 (29.8%)	8 (53.3%)	1 (reference)		1 (reference)	

OR: odds ratio; 95% CI: 95% confidence interval; *n*: frequency, ^∗^*p* value with this superscript was statistically significant (*p* < 0.05).

## Data Availability

The data used to support the findings of this study are available from the corresponding author upon request.
